# Copeptin, Routine Laboratory Parameters, and Ischemic Etiology of Heart Failure Predict Outcomes in Elderly Patients with Decompensated Heart Failure

**DOI:** 10.3390/biomedicines13092048

**Published:** 2025-08-22

**Authors:** Paulina Nadziakiewicz, Wioletta Szczurek-Wasilewicz, Michał Jurkiewicz, Michał Skrzypek, Agnieszka Gorzkowska, Mariusz Gąsior, Bożena Szyguła-Jurkiewicz

**Affiliations:** 1 Student’s Scientific Society, 3rd Department of Cardiology, Faculty of Medical Sciences in Zabrze, Medical University of Silesia, 40-055 Katowice, Poland; 2 2nd Department of Cardiology and Angiology, Silesian Center for Heart Diseases, 41-800 Zabrze, Poland; 3 Department of Pharmacology, Faculty of Medicine, University of Opole, 45-040 Opole, Poland; 4 Department of Biostatistics, Faculty of Public Health in Bytom, Medical University of Silesia in Katowice, 40-055 Katowice, Poland; 5 Department of Neurology, School of Health Sciences, Medical University of Silesia in Katowice, 40-635 Katowice, Poland; 6 3rd Department of Cardiology, Faculty of Medical Sciences in Zabrze, Medical University of Silesia, 40-055 Katowice, Poland

**Keywords:** biomarkers, copeptin, heart failure, elderly patients

## Abstract

**Background**: Diagnosing and predicting outcomes in elderly patients with heart failure (HF) is challenging due to atypical symptoms and the limited value of natriuretic peptides, highlighting the need to search for new risk stratification biomarkers in this population. **Aim**: We aimed to analyze factors associated with the composite endpoint (all-cause mortality or decompensated HF-related hospitalization) within six months of follow-up in elderly patients with left ventricular systolic dysfunction and decompensated HF, with particular emphasis on copeptin concentration. **Methods**: This is a retrospective observational study based on prospectively collected data of 279 consecutive elderly patients hospitalized between 2018 and 2023 due to decompensated HF. Inclusion criteria were age > 65 years, history of HF diagnosed at least two years before the index hospitalization, and left ventricular ejection fraction < 40% on admission echocardiography. Serum copeptin levels were measured using an Enzyme-Linked Immunosorbent Assay (ELISA) (Human Copeptin ELISA kit, Sunred Biological Technology Co, Shanghai, China). The primary endpoint was all-cause mortality or decompensated HF-related hospitalization during the six-month follow-up. **Results**: The median age of the study population was 77 years (IQR: 69–79), and 221 (79.2%) were male. The composite endpoint occurred in 110 patients (38.1%). Multivariable analysis showed that serum concentrations of copeptin [hazard ratio (HR) 1.053 (1.042–1.064), *p* < 0.0001], bilirubin [HR 1.085 (1.057–1.114), *p* < 0.0001], uric acid [HR 1.005 (1.003–1.006), *p* < 0.0001], high-sensitivity C-reactive protein (hs-CRP) [HR 1.208 (1.088–1.342), *p* < 0.0001], and sodium [HR 1.111 (1.025–1.203), *p* = 0.01], as well as ischemic etiology of HF [HR 3.969 (2.396–6.575), *p* < 0.0001], were independently associated with worse outcomes. **Conclusions**: Our study demonstrated that higher concentrations of copeptin, bilirubin, hs-CRP, and uric acid, as well as lower sodium levels and ischemic etiology of HF, were independently associated with all-cause mortality or HF-related hospitalization during a six-month follow-up in elderly patients with decompensated HF.

## 1. Introduction

Heart failure (HF) is the final stage of many cardiovascular diseases and is associated with high morbidity and mortality. With increasing life expectancy and advances in interventional and pharmacological treatments, the population of elderly patients with advanced HF is gradually increasing. Among patients hospitalized with decompensated HF, more than 50% are over 75 years of age [1,2]. In this group, clinical symptoms of HF in the elderly are often atypical and reflect not only cardiac dysfunction but also the impact of multiple comorbidities [2]. Most clinical trials evaluate much younger cohorts, and older patients are underrepresented in clinical registries, despite the growing population of elderly patients with HF. Furthermore, guideline-directed medical therapies are largely based on younger populations, and their findings may not be directly applicable to elderly patients, who differ substantially in clinical profile, multimorbidity, and prognosis [2,3].

In clinical practice, optimal management and accurate risk stratification in elderly HF patients remain challenging [1,2,4]. It seems that an integrated approach using multiple biomarkers reflecting different pathophysiological pathways of HF may improve risk stratification [5,6,7,8]. One of the promising indicators in HF is copeptin, which is a stable C-terminal fragment of provasopressin that plays a crucial role in the regulation of the water and electrolyte balance [9,10]. Elevated copeptin levels have been linked to greater disease severity, reduced cardiac index, and an increased risk of death or rehospitalization [9,10,11,12]. Moreover, the combination of copeptin with other biomarkers, such as N-terminal pro b-type natriuretic peptide (NT-proBNP), may further improve risk stratification in patients with HF [9,10,11]. However, the utility of copeptin in elderly patients is not fully understood. This study aims to identify factors associated with a composite endpoint of death or HF decompensation, with a particular focus on copeptin, in elderly patients with decompensated HF during a 6-month follow-up.

## 2. Materials and Methods

The research was conducted in accordance with the Declaration of Helsinki, and the study protocols were approved by the Ethics Committee of Medical University of Silesia (specific ethics codes KNW/0022/KB1/53/18, date of approval 19 June 2018; PCN/0022/KB1/20/I/21, date of approval 4 May 2021; PCN/CBN/0052/KB1/20/II/21/22, date of approval 20 September 2022). All participants provided written informed consent prior to inclusion in the study.

This is a retrospective observational study based on prospectively collected data of 279 consecutive elderly patients hospitalized between 2018 and 2023 due to decompensated HF. The inclusion criteria were age > 65 years, HF diagnosed at least two years prior to the index hospitalization, and left ventricular ejection fraction (LVEF) < 40% on admission echocardiography. The exclusion criteria included recent infection, irreversible kidney disease (eGFR < 30 mL/min/1.73 m^2^), chronic liver disease, malignancies, cerebrovascular accidents, and hematologic and autoimmune diseases. In all patients, physical examinations, clinical characteristics, anthropometric measurements, laboratory tests, and echocardiography were performed during the index hospitalization.

Peripheral venous blood samples were collected after 12 h of fasting at the time of enrollment. Laboratory assessments were performed using validated automated analyzers. Hematological indicators were determined with Sysmex XS-1000i or XE-2100 systems (Sysmex Corporation, Kobe, Japan). Biochemical parameters—including serum creatinine, total bilirubin, albumin, transaminases, glucose, and NT-proBNP—were assessed using a COBAS 6000 analyzer (Roche Diagnostics, Basel, Switzerland), while high-sensitivity C-reactive protein (hs-CRP) was measured via latex-enhanced immunoassay on the Cobas Integra 70 platform (Roche Diagnostics, Basel, Switzerland). NT-proBNP levels were measured with the Elecsys 2010 immunoassay system (Roche Diagnostics).

The Enzyme-Linked Immunosorbent Assay (ELISA) technique was used to measure copeptin serum levels (Human Copeptin ELISA kit, Sunred Biological Technology Co, Shanghai, China).

Patient deaths were verified based on medical records and the official registry of the National Health Fund. During the 6 months from inclusion in the study, patients were followed for HF decompensation and all-cause mortality. All laboratory and follow-up data were complete, and no patients were lost to follow-up.

### Statistical Analysis

Categorical variables were expressed as frequencies and percentages (%) and were compared between groups using Pearson’s chi-square test. Continuous variables were presented as median (interquartile range [IQR]) or mean (standard deviation), and comparisons between groups were made using the Mann–Whitney U test or Student’s *t*-test, depending on the distribution of the data. Normality of distribution was assessed using the Shapiro–Wilk test.

The Cox proportional hazards model was utilized to compute the hazard ratio (HR) and the 95% confidence interval (CI) for the composite endpoint between the analyzed groups. Cox’s univariable proportional hazard analysis was used to select potential independent predictive factors of the composite endpoint for inclusion in the multivariable analysis. The examined covariates included age, bilirubin, creatinine, highly sensitive C-reactive protein (hs-CRP), fibrinogen, uric acid, copeptin, body mass index, albumin and sodium, the presence of dyslipidemia and diabetes mellitus type 2, ischemic etiology, LVEF, and left ventricular end-diastolic dimension. The relationships between explanatory variables were calculated by the Spearman rank correlation coefficient, and multicollinearity was evaluated using tolerance and variance inflation factors. HRs were calculated, and the findings were presented with 95% confidence intervals (CIs). Receiver Operating Characteristic (ROC) curve analysis was performed to assess the predictive ability of independent variables identified in the multivariable analysis. The area under the curve (AUC) was calculated for each parameter to evaluate its discrimination power. Optimal cut-off values were determined using the Youden index, and corresponding sensitivity and specificity were reported. A *p*-value of <0.05 was considered statistically significant. All data analyses were conducted using SAS software version 9.4.

## 3. Results

Baseline characteristics of the population are shown in Table 1. The median age of the entire population was 77 years [IQR: 69–79], and 79.2% were male. During the 6-month follow-up period, the composite endpoint occurred in 110 patients (39.4%). All-cause mortality was 14.7% (n = 41), while 69 patients (24.7%) experienced episodes of HF decompensation.

Patients who met the composite endpoint were older, more likely to be classified as New York Heart Association (NYHA) class IV, and had a higher prevalence of comorbidities (type 2 diabetes and chronic obstructive pulmonary disease) than patients without the composite endpoint.

The baseline characteristics of pharmacological and device therapy are presented in Table 2.

Multivariable Cox proportional hazards analysis demonstrated that copeptin [HR 1.053 (1.042–1.064), *p* < 0.0001], bilirubin [HR 1.085 (1.057–1.114), *p* < 0.0001], uric acid [HR 1.005 (1.003–1.006), *p* < 0.0001], CRP [HR 1.208 (1.088–1.342), *p* < 0.0001], and sodium [HR 1.111 (1.025–1.203), *p* = 0.01] serum concentrations, as well as ischemic etiology of HF [HR 3.969 (2.396–6.575 *p* < 0.0001], are independently associated with the composite endpoint.

The results of the univariable and multivariable analyses are presented in Table 3.

**Figure 1:**
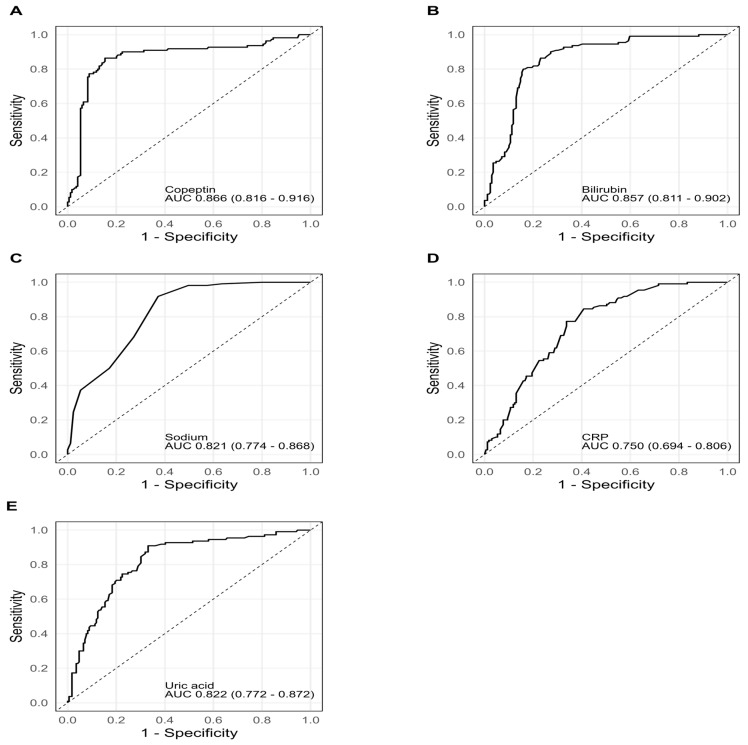
ROC curves for copeptin (**A**), bilirubin (**B**), sodium (**C**), hs-CRP (**D**), and uric acid (**E**).

ROC curve analysis demonstrated that copeptin had the highest prognostic value (AUC = 0.866; 95% CI: 0.816–0.916; *p* < 0.001), followed by bilirubin (AUC = 0.857; 95% CI: 0.811–0.902; *p* < 0.001), sodium (AUC = 0.821; 95% CI: 0.774–0.868; *p* < 0.001), uric acid (AUC = 0.822; 95% CI: 0.772–0.872; *p* < 0.001), and hs-CRP (AUC = 0.750; 95% CI: 0.694–0.806; *p* < 0.001). The ROC curves are shown in Figure 1A–E, and a summary is provided in Table 4.

## 4. Discussion

This single-center study demonstrated that higher copeptin, bilirubin, hs-CRP, and uric acid concentrations, as well as lower sodium concentrations and ischemic etiology of HF, are associated with all-cause mortality or rehospitalization during a six-month follow-up in elderly patients with decompensated HF. Among the analyzed parameters, copeptin demonstrated the highest prognostic value for predicting the composite endpoint in this population. However, copeptin should be considered an important component of the risk model rather than a standalone predictor, as it contributes to overall prognostic accuracy in combination with other clinical and laboratory parameters.

Our study provides new data on the utility of copeptin in assessing the composite endpoint over a six-month follow-up period in elderly patients with decompensated HF. Previous studies have shown that higher copeptin concentrations are associated with a worse prognosis in different groups of patients with HF [12,13,14]. However, the data regarding the clinical significance of copeptin in elderly patients with decompensated HF have been limited [15,16]. Alehagen et al. showed that elevated copeptin levels, along with the combination of elevated copeptin and NT-proBNP levels, were associated with an increased risk of all-cause mortality in elderly patients with HF during the long-term follow-up [15]. Holmström et al. also demonstrated that copeptin levels are elevated in elderly patients with normal LVEF and systolic HF [16]. However, the populations analyzed in those studies [15,16] differed from ours in several aspects. Their cohorts included younger patients, and only a small proportion had advanced HF with LVEF < 30% in NYHA classes III–IV. In contrast, our analysis focused exclusively on patients with end-stage HF with a median LVEF of 25% in NYHA classes III and IV. Copeptin reflects activation of the vasopressin system in response to hemodynamic stress and neurohormonal imbalance in HF, and its elevation is associated with the severity of the disease [17,18,19,20]. In elderly patients, impaired renal function, diminished baroreceptor sensitivity, and comorbidities may further enhance copeptin secretion [19,20,21,22], especially during an episode of decompensation [23,24]. In addition, impaired regulation of fluid volume due to a decrease in the kidneys’ ability to concentrate urine increases the dependence on vasopressin in maintaining water–electrolyte homeostasis [21].

Our study also confirms the prognostic importance of several other well-known risk factors in this elderly HF population. Lower sodium concentrations, as well as higher hs-CRP, bilirubin, and uric acid concentrations, were associated with all-cause mortality or rehospitalization during the six-month follow-up in the elderly patients with decompensated HF.

Hyponatremia is a common marker of HF decompensation and is associated with poor prognosis in HF, regardless of the patient’s age group [25,26,27,28]. In the elderly, reduced GFR and impaired intrarenal prostaglandin synthesis also limit water excretion [28,29], whereas diuretic use contributes to dilutional hyponatremia and fluctuations in water–electrolyte balance [29,30]. Hyponatremia is also an indirect marker of elevated AVP activity, which can be reflected by copeptin levels [31].

Another factor associated with the composite endpoint in our analysis was hs-CRP, which reflects smoldering inflammation in HF. Previous studies have also shown a strong association of hs-CRP with the severity of disease, worse prognosis, and decompensation of HF [32,33,34]. CRP levels increase during HF decompensation, reflecting systemic inflammation. The exact mechanism of the smoldering systemic inflammation in HF is not fully understood. However, studies suggest that it may increase microvascular endothelial inflammation and promote myofibroblast formation and interstitial collagen deposition, leading to adverse cardiac remodeling and progression of HF [33,34,35]. Moreover, comorbidities such as diabetes, hypertension, and COPD are common in the elderly population and induce a systemic pro-inflammatory state, which damages other cells, creating a positive feedback loop [36]. Although routine hs-CRP testing is not currently recommended in guidelines, our findings, in line with previous research, suggest its potential utility in evaluating acute HF patients.

Elevated serum bilirubin concentration was also associated with the composite endpoint in elderly patients with decompensated HF. Previous studies demonstrated that higher serum bilirubin levels are a prognostic indicator in elderly patients with decompensated HF [37,38,39]. Congestive hepatopathy is a common symptom of decompensated HF, occurring in ~70% of cases [37]. Among hepatic markers of congestive hepatopathy secondary to decompensated HF, the most sensitive is a slightly elevated serum bilirubin concentration [40]. From a pathophysiological point of view, hyperbilirubinemia in acute HF is mainly due to venous congestion, leading to passive liver congestion and, consequently, impaired hepatocyte function [41,42], compounded by hepatic hypoxia from reduced cardiac output, which further raises bilirubin levels [41,43]. Thus, elevated bilirubin level reflects high central venous pressure and reduced cardiac index, both signs of advanced HF [38].

Another independent factor of the composite endpoint in the analyzed elderly population with decompensated HF was elevated uric acid concentration, whose role as a cardiovascular risk factor has been extensively discussed for decades. Multiple large population-based studies have shown that increased uric acid levels were independent predictors of mortality in decompensated HF [36,44,45]. Uric acid, through the activation of pro-inflammatory pathways, stimulates the proliferation of vascular smooth muscle cells and endothelial fibrosis, and it also contributes to insulin resistance [36,44]. These mechanisms indirectly influence the development and progression of HF [36,42,44].

The last factor associated with rehospitalization for HF or death during the 6-month follow-up was ischemic etiology of HF, which is a well-known risk factor of worse outcomes in HF [46,47,48,49]. In European countries, myocardial ischemia is the leading cause of HF. It plays a significant role in myocardial remodeling, which encompasses changes in the heart’s shape and size, as well as diminished blood flow to the myocardium and a reduction in ejection fraction [48,49]. In addition, myocardial ischemia can both cause and accelerate HF decompensation [47,48]. However, further studies are needed to elucidate the mechanisms underlying the association of HF etiology with outcomes in HF patients who are admitted to the hospital with acute decompensated HF.

This study has several limitations. Firstly, it was a single-center analysis with a relatively small sample size, which may limit the generalizability of the findings to the entire population. Secondly, copeptin was measured only at admission, and we did not assess serial changes. Probably, dynamic changes in copeptin over time might provide additional prognostic information. Thirdly, our cohort consisted of very elderly patients with advanced HF, so the results may not be fully applicable to younger populations. Importantly, women were significantly underrepresented in the study cohort (approximately 20% of the enrolled population), which limits the applicability of the findings to older female patients and highlights the need for future studies addressing sex-specific differences in biomarker performance and prognosis. Finally, our study was observational in nature; therefore, it cannot establish causality between elevated copeptin levels and adverse outcomes.

## 5. Conclusions

Our findings highlight the utility of copeptin and other biochemical markers as potential tools for risk stratification in elderly patients with decompensated HF. However, future large multi-center studies are needed to validate these findings and determine whether the routine use of copeptin and other biomarkers can improve clinical outcomes or guide therapy in decompensated HF in the elderly.

## Figures and Tables

**Table 1:** Baseline characteristics of the study population.

	All Included Patients N = 279	Without Composite Endpoint * N = 169 (60.6%)	With Composite Endpoint * N = 110 (39.4%)	*p*
Baseline data				
Age, years	77.0 (69.0–79.0)	72.0 (68.0–77.0)	79.0 (77.0–81.0)	<0.0001
Male, n (%)	221 (79.2)	132 (78.1)	89 (80.9)	0.5729
Ischemic etiology of HF, n (%)	156 (55.9)	70 (41.4)	86 (78.2)	<0.0001
BMI, kg/m^2^	26.6 (23.8–29.5)	27.1 (23.8–30.4)	26.1 (24.3–28.1)	0.0376
NYHA III, n (%)	123 (44.1)	105 (62.1)	18 (16.4)	<0.0001
NYHA IV, n (%)	156 (55.9)	64 (37.9)	92 (83.6)	<0.0001
Comorbidities				
Hypertension, n (%)	185 (66.3)	106 (62.7)	79 (71.8)	0.1162
Type 2 diabetes, n (%)	144 (51.6)	66 (39.1)	78 (70.9)	<0.0001
Persistent AF, n (%)	138 (49.5)	83 (49.1)	55 (50.0)	0.8848
COPD, n (%)	70 (25.1)	35 (20.7)	35 (31.8)	0.0365
Laboratory parameters				
Copeptin, pmol/L	4.66 (3.05–8.77)	3.46 (2.28–4.58)	9.11 (7.36–11.80)	<0.001
WBC, ×10^9^/L	7.3 (6.1–8.4)	6.58 (5.40–7.92)	5.6 (4.70–6.73)	0.0007
Platelets, ×10^9^/L	194.0 (171.0–225.0)	179.0 (152.0–210.00)	157.5 (143.0–206.0)	0.0048
Hemoglobin, mmol/L	8.8 (8.2–9.7)	8.8 (8.3–9.7)	8.9 (8.2–10.0)	0.5572
Glucose, mmol/L	5.7 (5.3–6.1)	5.6 (5.2–6.1)	5.8 (5.4–6.1)	0.2183
HbA1c, %	5.8 (5.4–6.3)	5.8 (5.5–6.3)	5.8 (5.4–6.4)	0.5946
Creatinine, µmol/L	117.0 (90.0–134.0)	98.0 (87.0–126.0)	125.0 (111.0–146.0)	<0.0001
GFR, mL/min/1.73 m^2^	58.5 (50.3–71.4)	65.1 (54.0–78.6)	53.9 (45.1–60.1)	<0.0001
Total bilirubin, µmol/L	22.9 (17.3–28.6)	19.4 (13.3–23.0)	27.8 (25.1–32.2)	<0.0001
Albumin, g/L	44.0 (41.0–46.0)	44.0 (41.0–46.0)	45.0 (41.0–47.0)	0.0946
Uric acid, µmol/L	440.0 (378.0–515.0)	400.5 (341.0–465.00)	512.00 (433.0–603.0)	<0.0001
Urea, µmol/L	7.7 (5.8–11.3)	7.6 (5.9–11.9)	7.7 (5.7–11.3)	0.6649
Fibrinogen, mg/dL	329.0 (380.0–436.0)	347.0(306.0–398.00)	420.0 (345.0–51.0)	<0.0001
AST, U/L	32.0 (26.0–35.0)	31.0 (23.2–34.0)	30.0 (21.0–35.0)	0.8003
ALT, U/L	25.0 (20.0–33.0)	23.0 (18.1–31.5)	29.0 (19.0–33.1)	0.0598
ALP, U/L	79.0 (64.0–93.0)	78.0 (62.0–97.0)	80.5 (72.0–91.0)	0.1266
GGTP, U/L	80.0 (49.0–125.0)	71.0 (43.0–121.0)	89.0 (75.0–132.0)	0.0081
Cholesterol, mmol/L	4.3 (3.9–4.9)	4.2 (3.8–4.8)	4.5 (4.1–5.3)	<0.0001
hs-CRP, mg/L	2.3 (1.9–5.1)	2.95 (1.88–4.36)	4.5 (3.76–5.60)	<0.0001
Sodium, mmol/L	135.0 (133.0–138.0)	137.0(134.0–139.0)	133.5 (132.0–135.0)	<0.0001
NT-proBNP, pg/mL	4832 (3491.0–7191.0)	4625.0 (3500.0–7089.0)	5563.0 (3491.0–7191.0)	0.6488
Echocardiographic parameters				
LA, mm	45.0 (45.0–50.0)	45.0 (45.0–49.0)	45.0 (45.0–50.0)	0.7206
RVEDd, mm	35.00 (32.0–38.0)	35.0 (31.0–37.0)	35.0 (32.0–39.0)	0.0563
LVEDd, mm	75.0 (70.0–78.0)	71.0 (69.0–77.0)	77.0 (75.0–79.0)	<0.0001
LVEF, %	25.0 (23.0–30.0)	27.0 (25.0–30.0)	23.5 (20.0–26.0)	<0.001

* Death or HF decompensation within 6 months of inclusion in the study. Data are presented as medians (25th–75th percentile) or numbers (percentage) of patients. Abbreviations: ACEI, angiotensin-converting enzyme inhibitor; AF, atrial fibrillation; ALP, alkaline phosphatase; ALT, alanine aminotransferase; AST, aspartate aminotransferase; BMI, body mass index; COPD, chronic obstructive pulmonary disease glomerular filtration rate; GGTP, gamma-glutamyl transpeptidase; HbA1c, glycated hemoglobin; HF, heart failure; hs-CRP, high-sensitivity C-reactive protein; LA, left atrium; LVEDd, left ventricular end-diastolic dimension; LVEF, left ventricular ejection fraction; NT-proBNP, N-terminal pro-B-type natriuretic peptide; NYHA, New York Heart Association; RVEDd, right ventricular end-diastolic dimension; WBC, white blood cell.

**Table 2:** Baseline characteristics of pharmacological and device therapy.

	All Included Patients N = 279	Without Composite Endpoint * N = 169 (60.6%)	With Composite Endpoint * N = 110 (39.4%)	*P*
B-blockers, n (%)	219 (78.5)	139 (82.2)	80 (72.7)	0.05
Bisoprolol, mg/day	5.00 (2.50–5.00)	5.00 (2.50–5.00)	5.00 (5.00–5.00)	0.13
Nebivolol, mg/day	5.00 (2.50–5.00)	2.50 (2.50–5.00)	5.00 (2.50–5.00)	0.6
Carvedilol, mg/day	25.00 (25.00–25.00)	25.00 (25.00–25.00)	25.00 (12.50–25.00)	0.2
Metoprolol succinate, mg/day	90.00 (47.50–90.00)	90.00 (47.50–90.00)	90.00 (47.50–90.00)	0.4
ACEI, n (%)	148 (53)	89 (52.7)	59 (53.6)	0.87
Ramipril, mg/day	5.00 (2.50–5.00)	5.00 (2.50–5.00)	3.75 (2.50–5.00)	0.2
Perindopril, mg/day	5.00 (2.50–5.00)	5.00 (2.50–5.00)	5.00 (2.50–5.00)	0.3
ARB, n (%)	51 (18.3)	37 (21.9)	14 (12.7)	0.05
Valsartan, mg/day	80.00 (80.00–160.00)	80.00 (80.00–160.00)	80.00 (80.00–160.00)	>0.9
Telmisartan, mg/day	40.00 (40.00–40.00)	40.00 (30.00–40.00)	40.00 (40.00–40.00)	0.2
ARNI, n (%)	56 (20.1)	27 (16)	29 (26.4)	0.03
Sacubitril/valsartan, sacubitril, mg/day	48.60 (24.30–48.60)	48.60 (24.30–48.60)	48.60 (48.60–48.60)	0.4
Sacubitril/valsartan, valsartan, mg/day	51.40 (25.70–51.40)	51.40 (25.70–51.40)	51.40 (51.40–51.40)	0.5
Loop diuretics, n (%)	273 (97.8%)	168 (99.4%)	105 (95.5%)	0.037
Furosemide ^#^, mg/day	80.00 (80.00–160.00)	90.00 (80.00–160.00)	80.00 (40.00–120.00)	0.062
MRA, n (%)	101 (36.2)	89 (52.7)	12 (10.9)	<0.001
Spironolactone/eplerenone, mg/day	25.00 (25.00–50.00)	25.00 (12.50–50.00)	25.00 (25.00–50.00)	0.4
SGLT2, n (%)	129 (46.2)	63 (37.3)	66 (60)	0.0002
NOAC, n (%)	141 (50.5)	82 (48.5)	59 (53.6)	0.40
Digoxin, n (%)	76 (27.2)	39 (23.1)	37 (33.6)	0.05
Statin, n (%)	167 (59.9)	101 (59.8)	66 (60)	0.96
ASA, n (%)	95 (34.1)	66 (39.1)	29 (26.4)	0.03
ICD/CRT-D, n (%)	238 (85.3)	144 (85.2)	94 (85.5)	0.95

* Death or HF decompensation within 6 months of inclusion in the study. Data are presented as numbers (percentages) of patients. ^#^ Diuretic doses have been converted to furosemide equivalents (10 mg of torasemide = 40 mg of furosemide). Abbreviations: ACEI, angiotensin-converting enzyme inhibitor; ASA, acetylsalicylic acid; ARB, angiotensin receptor blocker; ARNI, angiotensin-receptor neprilysin inhibitor; CRT-D, cardiac resynchronization therapy-defibrillator; ICD, implantable cardioverter-defibrillator; MRA, mineralocorticoid receptor antagonist; NOAC, non-vitamin K antagonist oral anticoagulant; SGLT2, sodium–glucose transport protein 2.

**Table 3:** Univariable and multivariable analyses of factors.

	Univariable Analysis		Multivariable Analysis	
Parameter	HR (95% CI)	*p*	HR (95% CI)	*p*
Age	1.317 [1.239–1.400]	<0.0001		
Bilirubin	1.119 [1.096–1.144]	<0.0001	1.085 [1.057–1.114]	<0.0001
Creatinine	1.019 [1.012–1.025]	<0.0001		
CRP	1.357 [1.252–1.470]	<0.0001	1.208 [1.088–1.342]	<0.0001
Fibrinogen	1.011 [1.009–1.013]	<0.0001		
Uric acid	1.006 [1.005–1.008]	<0.0001	1.005 [1.003–1.006]	<0.0001
Copeptin	1.025 [1.016–1.035]	<0.0001	1.053 [1.042–1.064]	<0.0001
BMI ↓	1.067 [1.012–1.126]	0.017		
Albumin	1.052 [1.014–1.092]	0.007		
Sodium ↓	1.341 [1.246–1.444]	<0.0001	1.111 [1.025–1.203]	0.01
LVEDD	1.162 [1.115–1.213]	<0.0001		
EF ↓	1.238 [1.177–1.302]	<0.0001		
Ischemic etiology	3.738 [2.375–5.884]	<0.0001	3.969 [2.396–6.575]	<0.0001
Diabetes mellitus	2.858 [1.892–4.317]	<0.0001		

Abbreviations: see Table 1. ↓ per one unit decrease.

**Table 4:** Summary of the ROC curves analysis for selected biomarkers.

	AUC [±95 CI]	Cut-off	Sens. [±95 CI]	Spec. [±95 CI]
Copeptin	0.866 [0.816–0.916]	5.365	0.864 [0.785–0.922]	0.846 [0.783–0.897]
Bilirubin	0.857 [0.811–0.902]	24.7	0.800 [0.713–0.87]	0.84 [0.776–0.892]
Sodium	0.821 [0.774–0.868]	135	0.918 [0.85–0.962]	0.627 [0.55–0.7]
hs-CRP	0.75 [0.694–0.806]	3.4	0.845 [0.764–0.907]	0.592 [0.514–0.667]
Uric acid	0.822 [0.772–0.872]	425	0.909 [0.839–0.956]	0.669 [0.592–0.739]

Abbreviations: AUC, area under the curve; CI, confidence interval; see Table 1.

## Data Availability

The data presented in this study are available upon request from the corresponding author. The data are not publicly available due to privacy restrictions related to the rules in our institution.
